# Organochloride pesticides induced hepatic ABCG5/G8 expression and lipogenesis in Chinese patients with gallstone disease

**DOI:** 10.18632/oncotarget.9399

**Published:** 2016-05-17

**Authors:** Guixiang Ji, Cheng Xu, Haidong Sun, Qian Liu, Hai Hu, Aihua Gu, Zhao-Yan Jiang

**Affiliations:** ^1^ Department of Hepatobiliary and Pancreatic Surgery, Center of Gallbladder Disease, Shanghai East Hospital, Tongji University School of Medicine, Shanghai, China; ^2^ State Key Laboratory of Reproductive Medicine, Institute of Toxicology, Nanjing Medical University, Nanjing, China; ^3^ Key Laboratory of Modern Toxicology of Ministry of Education, School of Public Health, Nanjing Medical University, Nanjing, China; ^4^ Nanjing Institute of Environmental Sciences/Key Laboratory of Pesticide Environmental Assessment and Pollution Control, Ministry of Environmental Protection, Nanjing, China; ^5^ Department of Surgery, Shanghai Institute of Digestive Surgery, Ruijin Hospital, Shanghai Jiao Tong University School of Medicine, Shanghai, China

**Keywords:** gallstone disease, organochlorine pesticides, adipose tissue, liver, lipogenesis, Pathology Section

## Abstract

**Background:**

Organochlorine pesticides (OCPs) are one kind of persistent organic pollutants. Although they are reported to be associated with metabolic disorders, the underlying mechanism is unclear. We explored the association of OCPs with gallstone disease and its influence on hepatic lipid metabolism.

**Materials and Methods:**

OCPs levels in omentum adipose tissues from patients with and without gallstone disease between 2008 and 2011 were measured by GC-MS. Differences of gene expression involved in hepatic lipid metabolism and hepatic lipids content were compared in liver biopsies between groups with high and low level of OCPs. Using HepG2 cell lines, the influence on hepatic lipid metabolism by individual OCP was evaluated *in vitro*.

**Results:**

In all patients who were from non-occupational population, there were high levels of β-hexachlorocyclohexane (β-HCH) and p',p'-dichloroethylene (p',p'-DDE) accumulated in adipose tissues. Both β-HCH and p', p'-DDE levels were significantly higher in adipose tissues from patients with gallstone disease (294.3± 313.5 and 2222± 2279 ng/g of lipid) than gallstone-free controls (282.7± 449.0 and 2025±2664 ng/g of lipid, *P*< 0.01) and they were strongly related with gallstone disease (*P* for trend = 0.0004 and 0.0138). Furthermore, higher OCPs in adipose tissue led to increase in the expression of hepatic cholesterol transporters ABCG5 and G8 (+34% and +27%, *P*< 0.01) and higher cholesterol saturation index in gallbladder bile, and induced hepatic fatty acids synthesis, which was further confirmed in HepG2 cells.

**Conclusion:**

OCPs might enhance hepatic secretion of cholesterol into bile via ABCG5/G8 which promoting gallstone disease as well as lipogenesis.

## BACKGROUND

Organochlorine pesticides (OCPs) is one kind of persistent organic pollutants (POPs) that have been worldwide used in agricultural control of pests historically. Although OCPs are banned for agricultural use during the 70s and 80s last century, they still can be detected in water [1] or fat-containing foods such as fish, milk and meat [2]. They are characterized as high lipophilic and persistent in the environment, which are toxic to human health. They can be biomagnified in the food chain [3] and accumulate in the adipose tissues for many years. OCPs have prolonged half-life of years to decades and are resistant to degradation [4]. Consequentially, they can lead to long-term toxicity even after low dose of exposure [5].

Accumulating epidemiological evidences suggest an association between OCPs with disease related with metabolic disorders in glucose and lipids. Increased serum levels of OCPs are positively associated with increased prevalence of diabetes and metabolic syndrome [6-9], homeostasis model assessment of insulin resistance (HOMA-IR) [10-12] and cardiovascular disease[13]. However, the underlying mechanisms remain unclear yet. Among all the OCPs, more substantial evidences confirm positive association between higher p',p'-dichloroethylene (p', p'-DDE) level in adipose tissue and diabetes [14] and DDE [15] and hexachlorocyclohexane (HCH) [16] exposure are associated with body size in human. These studies collectively suggest the potential importance of OCPs leading to metabolic disorders in human.

Dyslipidemia are known to be risk factors for gallstone disease [17, 18], which is a common disease in western countries. Our recent survey in Shanghai city, China showed its incidence climbing up to 13.7% (Jiang ZY, et al. unpublished). Interestingly, in a previous case-control study, OCPs residues in serum were found to be higher in patients with gallstone disease in the area of Xiamen, China [19]. Since the serum OCPs levels are influenced by dietary lipids, they cannot reflect the burden of OCPs accumulation in body. However, the difficulties to obtain human adipose tissues, which can only be collected during surgical operation, make it a barrier to study the accumulation of OCPs in adipose tissue in association with certain diseases.

Whether OCPs lead to pathophysiological changes associated with gallstone disease through its effect on lipid metabolism in liver has never been investigated. In this study, OCPs levels were analyzed in a unique collection of omentum adipose tissues samples obtained during abdominal operation from patients with and without gallstone. In subgroups of gallstone patients with high and low OCPs levels, liver biopsy samples were also obtained in order to compare the hepatic expression of genes involving lipid metabolism between groups. Furthermore, the regulation of hepatic genes by OCPs was confirmed using HepG2 cell lines *in vitro*.

## RESULTS

### Characteristics of the study population and comparison of OCPs residues in adipose tissues between patients with and without gallstone disease

The characteristics of the gallstone and the gallstone-free patients are presented in Table 1. There were no significant differences in the age, gender distribution or BMI between two groups.

**Table 1:** Main characteristics of the subjects by gallstone status

Characteristic	Gallstone-free (*N* = 190)	Gallstone (*N* = 194)	*P*
Age (years, mean ± SD)	58.47 ± 16.02	56.38 ± 13.35	0.165 ^a^
Gender			0.269 ^b^
Males	87	78	
Females	103	116	
BMI (mean±SD)	24.37 ± 2.86	24.41 ± 3.33	0.8997 ^a^
HCB (ng/g of lipid)			0.2840 ^c^
Median	62.04	59.27	
Min–Max	2.732- 320.6	6.576-516.7	
Percentiles 10–90	35.48- 107.24	20.80-139.6	
*p,p'*-DDE (ng/g of lipid)			0.0080^c^
Median	1213	1542	
Min–Max	19.93-17261	12.83- 18135	
Percentiles 10–90	231.3-4108	29.06- 9991	
β-HCH (ng/g of lipid)			0.0006^c^
Median	92.01	233.5	
Min–Max	12.45- 3536	7.91-3088	
Percentiles 10–90	33.46 −805.4	33.50-622.3	

a Student's t test;

b chi-square test;

c Mann-Whitney's U-test.

**Figure 1:**
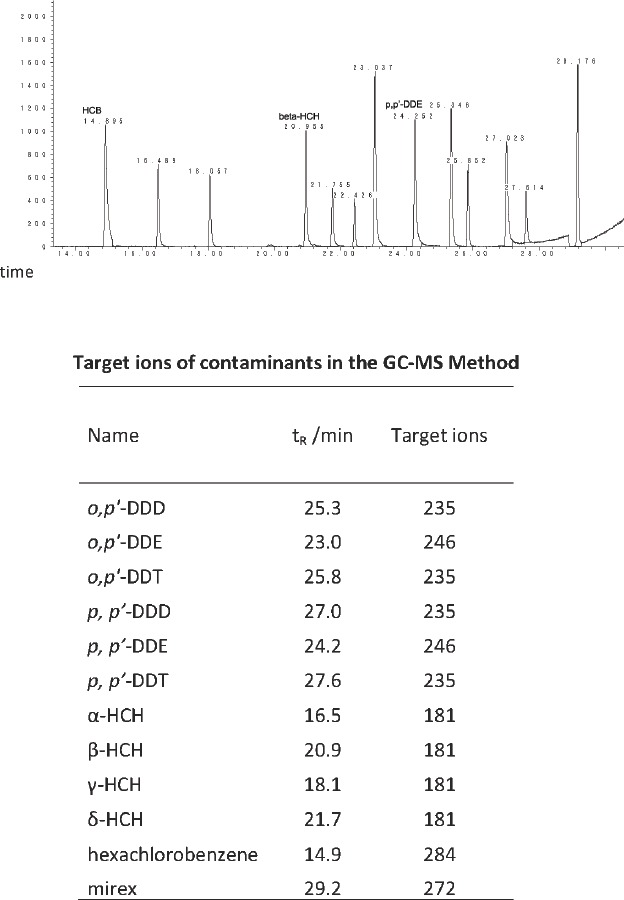
Chromatogram of 12 organochlorine pesticides (upper part) and the target ions of contaminants in the GC-MS method (lower part)

Among the 12 OCPs detected in adipose tissues (Figure 1), the detected percentage of HCB, β-HCH and p', p'-DDE were 100%, and 26.2% for α-HCH. For other types of OCPs, they were below the detection level in any of the subjects.

**Figure 2:**
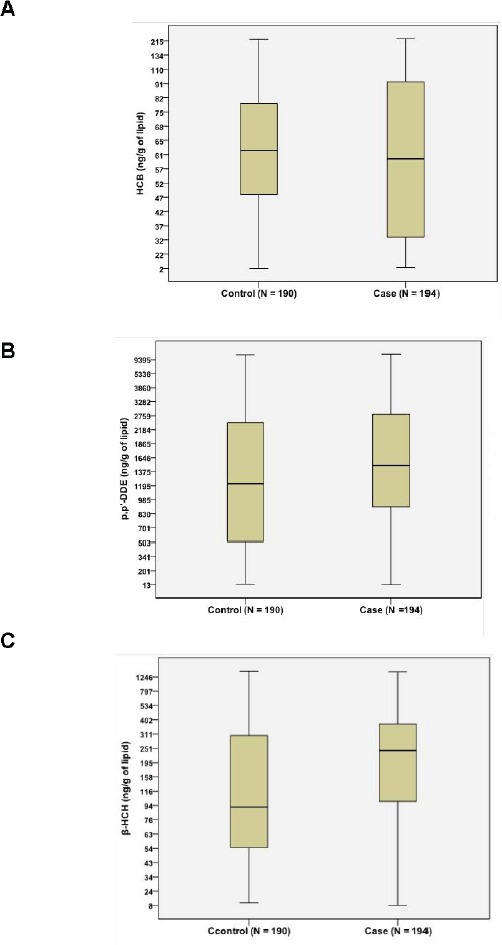
Comparison of OCPs between patients with gallstone disease (case) and gallstone-free controls (control) No difference was found for HCB **A.** between groups. Both β-HCH **B.** and p', p'-DDE **C.** content were significantly higher in gallstone patients (case) than in gallstone-free controls (control), *P* < 0.05.

The mean concentrations of p', p'-DDE and β-HCH were 2222 and 294.3 (ng/g of lipid) in patients with gallstone disease, and 2025 and 282.7 (ng/g of lipid) in gallstone-free patients (Table 1). p', p'-DDE and β-HCH concentrations in patients with gallstone disease were significantly higher than those in gallstone-free patients (*P* < 0.05), whereas the concentration of HCB showed no difference between the groups (Figure 2). Power analysis was > 90% at α = 0.05 level suggesting the satisfaction of sample size in the present study.

### High adipose OCPs levels positively associated with gallstone disease

Based on the fourth quartile of OCPs in all of the patients, quartile division was applied to divide the subjects into 4 subgroups for trend analysis and elucidation of the dose-response for gallstone risk of each OCP. After adjusted for age, gender and BMI, p', p'-DDE levels was significantly associated with gallstone risk, with ORs (95%CI) for the quartile 2 to quartile 4 of 3.49 (1.93-6.33), 2.38 (1.32-4.27) and 2.48 (1.38-4.46), respectively (p-trend = 0.0138, Table 2). A similar association was also found between β-HCH quartiles and the risk of gallstone [ORs (95%CI) for the 3rd and 4th quartiles of 3.70 (2.03-6.74) and 1.81 (1.02-3.23), respectively (p-trend = 0.0004), Table 2]. However, there was no association for HCB levels with gallstone disease.

**Table 2:** Risk of gallstone disease in association with organochlorine pesticides in adipose tissues

Compound/concentration (ng/g of lipid weight)	Control *N* = 190	Case *N* = 194	OR (95% CI)
	*N* (%)	*N* (%)	
HCB			
2.73-41.1	41 (21.6)	55 (28.4)	1.00
41.1-61.68	52 (27.4)	44 (22.7)	0.63 (0.36, 1.12)
61.68-84.62	49 (25.8)	47 (24.2)	0.72 (0.40, 1.26)
84.62-516.7	48 (25.3)	48 (24.7)	0.75 (0.42, 1.32)
*P*-value for trend	0.8254		
*p,p'*-DDE			
12.83-721.7	65 (34.2)	31 (16.0)	1.00
721.7-1351	36 (18.9)	60 (30.9)	3.49 (1.93, 6.33)*
1351-2558	45 (23.7)	51 (26.3)	2.38 (1.32, 4.27)*
2558-18135	44 (23.2)	52 (26.8)	2.48 (1.38, 4.46)*
*P*-value for trend	0.0138		
β-HCH			
7.91-59.18	58 (30.5)	38 (19.6)	1.00
59.18-174.8	61 (32.1)	35 (18.0)	0.86 (0.48, 1.54)
174.8-363.8	28 (14.7)	69 (35.6)	**3.70 (2.03, 6.74)**
363.8-3536	43 (22.6)	52 (26.8)	1.81 (1.02, 3.23)*
*P*-value for trend	0.0004		

a ORs adjusted for age, gender and BMI.

* *P* < 0.05 compared with the lowest pesticides concentration.

Correlations between OCPs and serum glucose and lipids were analyzed by multivariate logistic regression. Only a positive correlation between β-HCH and glucose was found after adjusted with age, gender and BMI (OR=1.215 95%CI: 1.006-1.468, P=0.04, Table 3).

**Table 3:** Multivariate analysis for β-HCH levels in human adipose tissue and biochemical index (n = 194)

Variables	Coef.^a^	Std. err.	95%Conf. interval	*P*-value
Blood glucose	0.195	0.096	0.006, 0.384	0.044
Total cholesterol	−0.020	0.074	−0.166, 0.126	0.786
Triglyceride	0.013	0.047	−0.080, 0.106	0.785
High density lipoprotein	−0.001	0.052	−0.103, 0.101	0.985
Low density lipoprotein	−0.057	0.108	−0.271, 0.157	0.599
ALT	−5.97	5.22	−16.3, 4.33	0.255
AST	−3.41	2.29	−7.93, 1.11	0.138
Apolipoprotein A^b^	−0.011	0.026	−0.063, 0.041	0.680
Apolipoprotein B^b^	−0.022	0.028	−0.076, 0.033	0.435
NEFA^b^	−0.005	0.030	−0.065, 0.055	0.874
Apolipoprotein E^b^	0.897	1.88	−2.82, 4.61	0.633

a Regression coefficients were adjusted for age, gender, and BMI;

b N = 163.

Abbrieviation: ALT: alanine aminotransferase; AST: aspartate aminotransferase; NEFA: non-esterified fatty acids.

### High OCPs level in adipose tissue contributed to disorders of hepatic lipids metabolism in patients

The main defect led to gallstone formation is hyper-secretion of cholesterol into bile by liver [17, 20]. We next investigated whether β-HCH and p', p'-DDE levels were related with disorders in hepatic lipid metabolism in gallstone patients. Liver biopsies were obtained from a subgroup of gallstone patients. The patients were classified into three quartiles according to the level of both β-HCH and p', p'-DDE in adipose tissue. The highest tertile of both OCPs was designated as high-level group and the lowest tertile as low-level group. Gene expression levels in the pathway of cholesterol, bile acids, phospholipids and fatty acids metabolism were measured.

**Figure 3:**
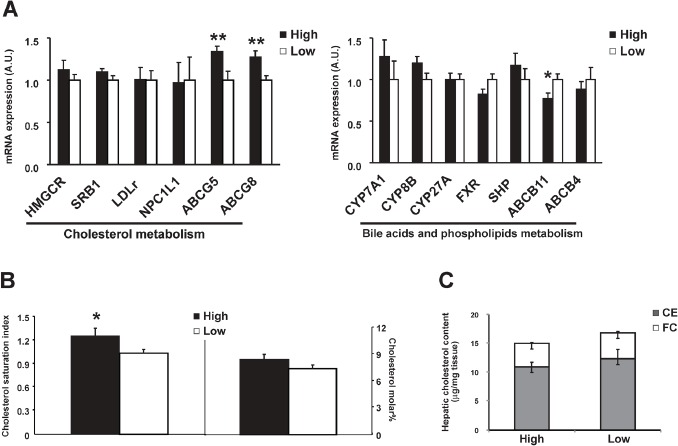
Comparison of hepatic gene expressions between high OCPs (p', p'-DDE and **β**-HCH) level group (*n* = 17) and low level group (*n* = 19) **A.** Genes involved in cholesterol, phospholipids and in bile acid metabolism; **B.** Cholesterol saturation index and cholesterol molar% in gallbladder bile; **C.** Free cholesterol and cholesteryl ester level in liver. Data were expressed as means ± SEM. ‘*' represents *P* < 0.05 and ‘**' represents *P* < 0.01.

ABCG8, together with ABCG5, forms heterodimers in the canalicular membrane of hepatocytes and are responsible for the secretion of hepatic cholesterol into bile [21, 22]. The mRNA expressions of both *ABCG5* and *ABCG8* were significantly higher in the high-level group (+34% and +27%, P<0.01, Figure 3A). This change was accompanied with higher cholesterol saturation index (CSI, P<0.05) in gallbladder bile in the high-level group compared with the low-level group as well as a trend of higher cholesterol molar% (Figure 3B and Table 4). Positive correlation was found for β-HCH in association with *ABCG5* (r=0.47)*, ABCG8* (r=0.35) expression and CSI (r=0.50), P<0.05, as well as for p', p'-DDE (r=0.47, 0.39 and 0.48, respectively, P<0.05). These results suggested that higher β-HCH and p', p'-DDE might promote hyper-secretion of biliary cholesterol which favors gallstone formation via hepatic ABCG5/G8. The genes involved in hepatic cholesterol synthesis or absorption did not differ (Figure 3A). The mRNA expressions of the key enzymes for hepatic bile acid synthesis were about the same between groups. The canalicular bile salt export pump, *ABCB11*, was 23% lower in high-level group (P<0.05, Figure 3A), suggesting the existence of decreased secretion of biliary bile salts in these patients as well.

**Table 4:** Comparison of biliary lipid composition between high-level OCPs group and low-level OCPs group (means ± SEM)

	High-level group	Low-level group
Cholesterol (molar%)	8.4±0.6	7.3±0.4
Bile acids (molar%)	70.5±1.6	68.1±2.8
Phospholipids (molar%)	21.8±1.5	24.7±2.7
Total lipids (g/L)	10.3±1.1	10.8±0.9
CSI	1.25±0.09^*^	1.03±0.05

* *P* < 0.05 when compared with low-level group by t-test

**Figure 4:**
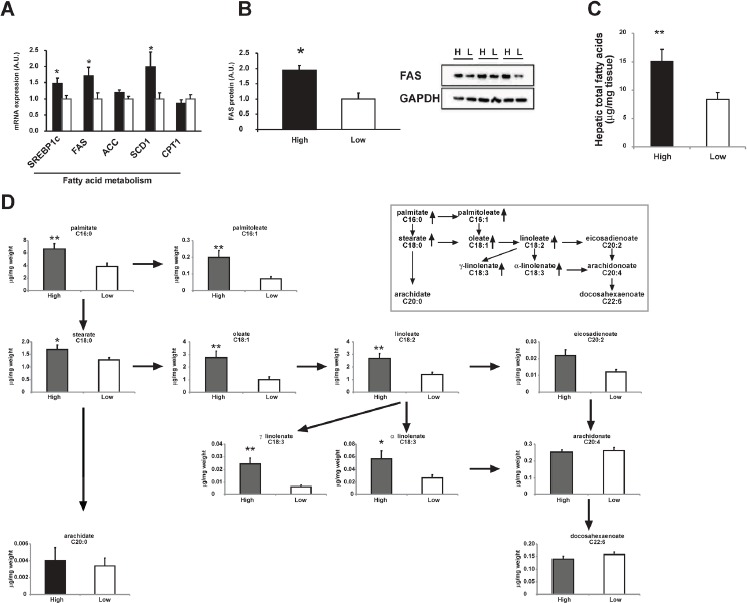
Comparison of hepatic fatty acid metabolism between high OCPs (p', p'-DDE and **β**-HCH) level group and low level group **A.** mRNA expression of genes involved in fatty acid metabolism. **B.** Protein level of FAS in pooled liver homogenates from high OCPs level group (H) and low level group (L). **C.** Hepatic total fatty acids content. **D.** Hepatic individual fatty acid contents. The pathway of main fatty acids was shown in the in-set figure. Data were expressed as means ± SEM. ‘*' represents *P* < 0.05 and ‘**' represents *P* < 0.01.

**Figure 5:**
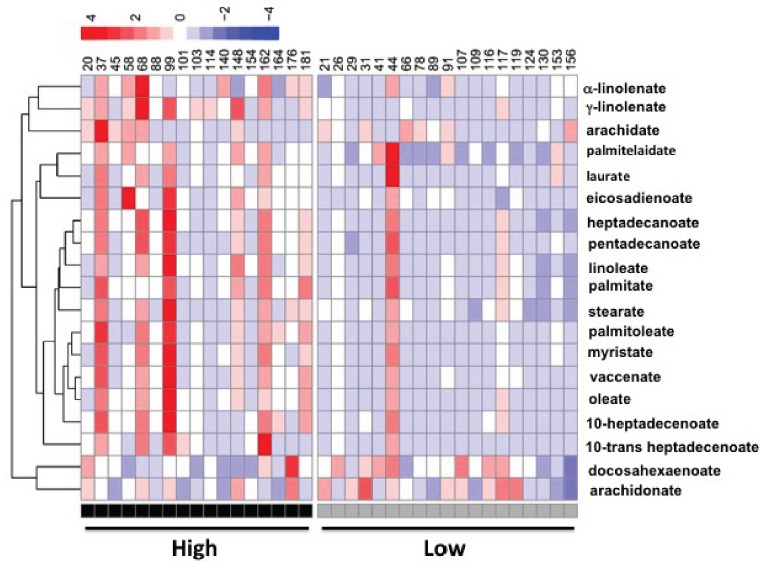
A heatmap display of quantitative analysis of all individual fatty acids in liver between patients with high OCPs (p', p'-DDE and **β**-HCH) level and low OCPs level

Interestingly, the key enzymes in fatty acid synthesis, fatty acid synthase (*FAS*) and stearoyl CoA desaturase 1 (*SCD1*), were 71% and 99% higher in the high-level group, respectively (Figure 4A) as well as the protein level of FAS (Figure 4B). Hepatic total fatty acids level was also significantly higher in the high-level group than the low-level group (Figure 4B) suggesting an enhanced lipogenesis in patients with high level of adipose OCPs. The differences in profile of individual fatty acid between the two groups were shown in Figure 4D. A more in-depth insight into patterns of individual fatty acids in high-level group and low-level group was shown in Figure 5. The increase of fatty acid synthesis might be activated by sterol response element binding protein 1c (SREBP1c) [23], which was 48% higher in the high-level group (Figure 4A).

Carnitine palmitoyl transferase 1 (CPT1), which is responsible for the fatty acid β-oxidation, did not differ between groups (Figure 4A). No difference was observed for free cholesterol or cholesteryl ester levels in liver either (Figure 3C), suggesting no excessive accumulation and esterification of cholesterol in liver.

### OCP induced genes involving lipid metabolism in HepG2

**Figure 6:**
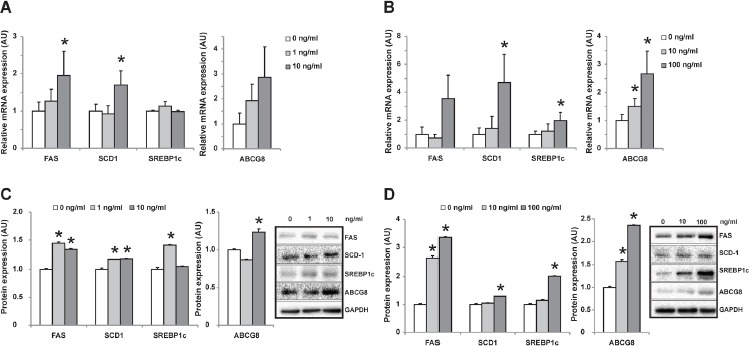
Changes of gene expression in HepG2 cells after incubated with individual OCP mRNA **A.** and protein expression **C.** of genes in HepG2 cells incubated with increased concentration of p', p'-DDE (0, 1, 10ng/mL). mRNA **B.** and protein expression **D.** of genes in HepG2 cells incubated with increased concentration of β -HCH (0, 10, 100ng/mL). ‘*' represents *P* < 0.05 as compared with the concentration at 0 ng/mL.

Because the co-existence of OCPs in human tissues, we incubated human hepatoma cell line – HepG2 with either β-HCH or p', p'-DDE separately to differentiate the role of each OCP. β-HCH induced FAS, SCD1 SREBP1c and ABCG8 expression in HepG2 cells dose-dependently (Figure 6B and 6D). Similar effect was observed when incubated with p', p'-DDE (Figure 6A and 6C). These data suggested β-HCH and p', p'-DDE could synergistically influence hepatic lipid metabolism of cholesterol and fatty acids.

## DISCUSSION

This study for the first time investigated the association of OCPs levels in omentum adipose tissues with gallstone disease and molecular changes in lipid metabolism in human liver. We found β-HCH and p', p'-DDE levels in adipose tissues were higher in patients with gallstone disease (Table 1 and Figure 2) and strongly positively related with gallstone disease (Table 2). Our results also showed that higher OCPs in adipose tissue led to increased expression of cholesterol transporters ABCG5/G8 in liver, higher cholesterol saturation index in gallbladder bile in patients (Figure 3), as well as increased hepatic lipogenesis (Figure 4 and 5). Both β-HCH and p', p'-DDE could induce the expression of genes involved in cholesterol excretion and fatty acid synthesis in HepG2 cells (Figure 6).

Due to their lipophilic property, OCPs can accumulate in adipose tissues and other organs. They are resistant to degradation in the body and can exert their influences persistently. However, only limited studies have measured the levels of OCPs in human adipose tissues collected from patients at different regions (Table 5): in breast adipose tissues [24-26] and in subcutaneous adipose tissue [16, 27, 28]. Though limited, these data collectively provide evidences for substantial accumulation of OCPs in non-occupational populations even long after banning of the usage of OCPs. The OCPs level in adipose tissue were fold-higher in our subjects from East China compared with data from Belgium, Canada and US, but less than that from Mexico or Poland. OCPs still can be detected in water [1] and the residual levels of OCPs in animal meats such as fish, beef, etc [29]. The levels of OCPs in adipose tissue may more reflect the burden of OCPs exposure in body and it has the advantage of less influence by dietary lipids than serum or plasma. Albeit, much attention has been paid concerning serum OCPs in association with various diseases such as cardiovasulcar disease, diabetes, metabolism syndrome [6-9] due to the difficulty in obtaining adipose tissue in the populations. Therefore, in this study, we collected omentum adipose tissue in patients and found strongly positive association between OCPs levels and gallstone disease.

**Table 5:** Comparison of OCPs levels in adipose tissues in subjects from different countries

No	Source	Country	year	number	Age(years)	HCB	β-HCH	DDE	References
**1**	Abdominal adipose tissue	Belgium	1998-2007	98	>18	N/A	19	205	Obesity 2011; 19: 709-714 a
**2**	Abdominal adipose tissue	Belgium	2000	20	19-77	46	N/A	280	Environ Res 2002; 88: 210 a
**3**	Breast adipose tissue	Canada	1995-1997	217 cases 213 controls	average: 57.7 vs 53.9	32.0 vs 30.1	43.1 vs 41.5	693 vs 596	Cancer Epidemiol Biomarker Prev 2000; 9: 55 a
**4**	Breast adipose tissue	USA	1994-1997	186 cases 304 controls	40-79	N/A	N/A	772.8 vs 789.5*	Am J Epidimiology 1999;150:453 a
**5**	Omentum adipose tissue	China, Shanghai	2008-2009	190 cases 190 controls	17-79	67.8	282	2025	The present study a
**6**	Breast adipose tissue	Mexico	NA	60	18-44	60	140	4360	Arch Environ Contam Toxicol 2001; 40: 432 a
**7**	Subcutaneous adipose tissue	Poland	1989-1992	277	10-80	310	228	5745	Bull Environ Contam Toxicol 1994; 52: 40 a
**8**	Serum	USA	1999-2004	4433	20-85	11.3	10.1	389.5	The NHANES survey b
**9**	Serum	China, Xiamen	2009-2010	150 cases 150 controls	average: 47.71 vs 48.35	N/A	2.881 vs 7.986	1.137 vs 0.677	Ann Epidemiol 2012; 22: 592 c
**10**	Serum	Shanghai, China	2013-2014	124 cases 109 controls	3-6	46.12 vs 22.86	111.11 vs 31.49	166.52 vs 6.97	Enviromental Res 2016; 46:125
**11**	Whole blood	Japan,	2002-2005	186	17-47	100	150	610	Science Total Environment 2012; 426: 73. d

a: unit=ng/g of lipid weight;

b: unit=ng/L;

c: μg/L;

d: pg/g wet mass of whole blood;

*: geometric means

Our finding that subjects with higher β-HCH and p', p'-DDE levels in adipose tissues were at higher risk of developing gallstone disease is in line with Su et al's [19]. More importantly, we found both mRNA of ABCG5 and ABCG8 were higher in patients with high OCPs level. Hepatic ABCG8 expression was induced by both β-HCH and p', p'-DDE in HepG2 cells *in vitro*. ABCG5/G8 are key players in regulating biliary cholesterol content [21, 22]. Increased hepatic expression of ABCG5 and ABCG8 mRNA in gallstone patients has been reported [30], which represents the main defect leading to hyper-secretion of cholesterol in hepatocyte. In the patients with high OCPs level, higher CSI in gallbladder was observed as well, suggesting the presence of an enhanced biliary secretion of cholesterol into bile by hepatocyte. Moreover, we found lower ABCB11 expression in the high-level group suggested presence of decreased biliary bile acid secretion by hepatocytes, even no difference in bile acid synthesis existed.

In this study, we also found defects in hepatic fatty acid synthesis patients with high-level of β-HCH and p', p'-DDE as evidenced by higher expression of FAS, SCD-1, increased hepatic total fatty acid and individual fatty acids contents (Figure 4 and 5). This was due to the activation of hepatic SREBP1c which is the key regulator of lipogenesis gene [23]. Mixed OCPs in high fat diet could induce hepatic SREBP1c expression in rats [31] and led to liver steatosis. In rats fed with high fat diet, p', p'-DDE exposure can induce liver levels of fatty acids as palmitic, stearic, oleic acids [32]. Using HepG2 cells, we also observed an induction of genes in lipogenesis by β−HCH and p', p'-DDE *in vitro* (Figure 5). These observations are consistent with what we found in human liver from patients with high OCPs levels.

**Figure 7:**
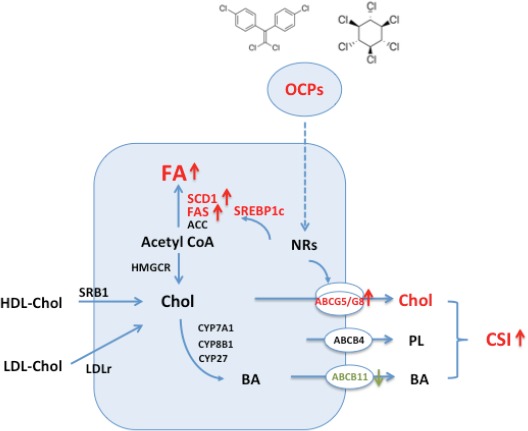
Schematic showing changes of hepatic lipid metabolism in hepatocyte exposed to high level of organochlorine pesticides (p', p'-DDE and β-HCH) (1) Increased hepatic canalicular cholesterol transporters ABCG5 and ABCG8 associated with higher biliary cholesterol saturation index; (2) Increased expression of key enzymes in fatty acid synthesis pathway and high level of hepatic total fatty acid content and fatty acid composition. Abbrieviations: ACC: acetyl-CoA carboxylase; ABC: ATP binding cassette; BA: bile acids; Chol: cholesterol; CSI: cholesterol saturation index; CYP7A1: cholesterol 7 α -hydroxylase, CYP8B1: cholesterol 12α-hydrolylase; CYP27: cholesterol 27-hydroxylase; FA: fatty acid; FAS: fatty acid synthase; HDL: high density lipoprotein; HMGCR: 3-hydroxy-3-methylglutaryl coenzyme A reductase; LDLr: low density lipoprotein receptor; NRs: nuclear receptors; OCPs: organochlorine pesticides; SCD1: stearoyl CoA desaturase 1; SREBP1c: sterol regulatory element biding protein 1c; SRB1: scavenger receptor B type 1.

In conclusion, our study provided important data showing the extent of OCPs accumulation in adipose tissue in non-occupational subjects living in East China and found strong association between high OCPs levels in adipose tissue and gallstone disease. The mechanistically molecular changes in hepatic lipid metabolism induced by OCPs were schematically shown in Figure 7. Due to the difficulty to obtain adipose tissue in human, such data are rare and may provide important insights for an understanding the potential chronic influences on hepatic metabolic homeostasis by OCPs. In general population, people are experienced background exposure to OCPs through food consumption. OCPs can accumulate in adipose tissue for decades and are resistant to degradation and such chronic low-level exposure seems not to be risk-free. Our present findings suggest the public health significance of environmental OCPs in relation with metabolic disorders in human.

## MATERIALS AND METHODS

### Patients and sample collection

About 200~800 mg great omentum adipose tissue samples were collected from 194 patients with cholesterol gallstone disease during laparoscopic cholecystectomy between May 2008 and December 2011. A wedge of about 0.2~0.5 gram liver biopsies were taken from the edge of right liver during the laparoscopic cholecystectomy in 60 patients with gallstone disease. Cholesterol gallstones were confirmed by visual inspection of the typical cut-surface of gallstones or, when necessary, by enzymatic cholesterol analysis. During the same period, great omentum adipose tissue from 190 gallstone-free patients (as controls) undergoing abdominal surgery unrelated to the gallstone disease (36% appendicitis surgery, 21% inguinal hernia, 16% liver and spleen rupture surgery, and 27% other surgery). All the controls were proved to be gallstone-free by B-type ultrasonography. All collected tissue samples were snap-frozen in liquid nitrogen, and then stored at −80°C until analysis. The study protocol conformed to the ethical guidelines of the Declaration of Helsinki and was approved by the Ethical Committee at Shanghai East Hospital, Tongji University School of Medicine and Shanghai Ruijin Hospital, Shanghai Jiaotong University School of Medicine. Written informed consent was obtained from each patient.

### Organochlorine analyses

#### Sample preparation and purification

The total adipose tissue sample was homogenized twice with 3 mL of acetonitrile (plus 1 mL of formic acid). All the homogenate (plus 1 gram of sodium chloride) was collected to centrifuge at 10,000 r/min for 5 min. The supernatant was cleaned by SPE PSA cartridges (Waters, USA) to remove the impurities, which was washed with 25 mL of acetonitrile- toluene (3:1). All the eluate was concentrated to about 1 mL by rotatory evaporator and dried by nitrogen. One mL of hexane was added to dissolve the pesticides and then the sample extraction solution was centrifuged at 14,000 r/min for 5 min at the temperature of 4°C. Then the supernatant mixed with the internal standard solution (heptachlor epoxide) was analyzed by GC-MS.

#### Instrumental analysis

The total of 12 OCPs were analyzed simultaneously by GC-MS using Agilent 7890A gas chromatograph, operating in EI mode. The final sample extract was injected onto a DB-1701 capillary column (30m×0.25mm×0.25μm, Agilent, USA) in the split mode (20:1) using helium as carrier gas at a constant flow rate of 1.2 mL/min. The temperature of the injector was 290°C. The oven temperature was programmed to warm up from 40°C (holding for 1min) to 130°C at a rate of 30°C /min, then to 250°C at a rate of 5°C / min, and then to 300°C (5min) at a rate of 10°C / min. Ionization energy was 70eV. The ion source temperature was 230°C and the quadruple rod temperature was 150°C. For each chemical, the target ions were monitored for quantification (Figure 1). The chromatogram of all the analytical contaminants was shown in Figure 1.

#### Quality control and assurance

The analytical method was validated and showed no interference in the retention time (t_R_) region of the test substances. The levels of quantification (LOQ) of all chemicals in the adipose tissue sample were between 2.50~19.0 ng/g, with recoveries between 72.8%~104.5%. The laboratory reagent, blank samples, and spiked samples, were treated and analyzed with the same method as the actual samples. The relative standard deviation (RSD) of all the controls was between 9.65%~16.8%, which showed that the method was stable.

### Cell culture

HepG2 cells were grown in DMEM supplemented with 10% FBS. Cells were plated in 35 mm dishes and reached ~70% confluence. After treated with p', p'-DDE (final concentration: 0, 1, 10 ng/ml) or β-HCH (final concentration: 0, 10, 100 ng/ml) for 24 hours, cells were collected. All experiments were performed in triplicates and repeated at least twice.

### Relative RNA expression level measurement

Total RNA of liver tissue or HepG2 cells was extracted with Trizol (Invitrogen, Calsbad, CA) and reverse-transcribed into cDNA. Real-time quantitative PCR for hepatic genes involved in lipid metabolism was performed in triplicates using SYBR-Green (Power Master Mix Sybr Green, Applied Biosystems, Foster City, CA). All the primer sequences are available on request. Data were calculated by the delta-Ct method using cyclophilin A as the internal control.

### Hepatic fatty acids and cholesterol content measurement

Hepatic fatty acids contents were measured as previously described [33, 34]. In brief, about 20 mg liver tissue was homogenized in ice cold PBS. Fatty acids were extracted by hexane and isopropanol. After incubated with methanol and sulfuric acid, the products, fatty acid methyl esters, were separated and identified by gas chromatography. Hepatic cholesterol concentration was assayed by gas chromatography mass spectrometry as previously reported [30].

### Analysis of biliary lipids composition

Biliary cholesterol, total bile acids, and phospholipids in gallbladder bile were measured as described previously [30]. The cholesterol saturation index (CSI) was calculated using Carey's critical table [35].

### Western blot

Liver homogenates or cell lysates were separated on SDS-PAGE gel and then transferred onto nitrocellulose membrane. After blocking in 5% non-fat dry milk in PBST, the membranes were incubated overnight at 4°C with specific primary antibody against FAS, SCD1, SREBP1c or ABCG8. After washing, secondary antibodies were incubated. The results were detected and recorded with Molecular Imager camera (Bio-Rad, Hercules, CA, USA) and densitometry analyses were performed for the quantification of results with GAPDH as a loading control.

### Statistical analyses

Data analysis was performed using SAS version 9.1 (SAS Institute Inc., Cary, NC, USA). Differences in select variables between groups were evaluated using the Student t test or *χ*^2^ test. The variables for concentrations of OCPs were evaluated using Mann-Whitney's U-test.

The OCPs concentrations were categorized into four groups, on the basis of percentile intervals <25%, 25% to <50%, 50% to <75%, and ≥75%. Logistic regression was performed to obtain the odds ratio (OR) for risk of gallstone disease across the categories of OCPs, adjusting for sex, age, and BMI using the lowest category (25%) as the reference group.

Multivariable linear regression was used to explore relationships between OCPs concentrations and biochemical index (e.g. serum concentrations of blood glucose, total cholesterol, triglyceride and *et al.*). Covariates considered for inclusion in the multivariate regression linear models included age, gender, and BMI. Age and BMI were modeled as continuous variables.

## Abbreviations

ACC: acetyl-CoA carboxylase;
ABC: ATP binding cassette;
BA: bile acids;
Chol: cholesterol;
CSI: cholesterol saturation index;
CYP7A1: cholesterol 7 α -hydroxylase;
CYP8B1: cholesterol 12α-hydrolylase;
CYP27: cholesterol 27-hydroxylase;
DE: p',p'-dichloroethylene;
FA: fatty acid;
FAS: fatty acid synthase;
HDL: high density lipoprotein;
HCH: β-hexachlorocyclohexane;
HMGCR: 3-hydroxy-3-methylglutaryl coenzyme A reductase;
LDLR: low density lipoprotein receptor;
NRs: nuclear receptors;
OCPs: organochlorine pesticides;
SCD1: stearoyl CoA desaturase 1;
SREBP1c: sterol regulatory element biding protein 1c;
SRB1: scavenger receptor B type 1.
